# Relationship of indoor solid fuel use for cooking with blood pressure and hypertension among the elderly in China

**DOI:** 10.1007/s11356-022-19612-1

**Published:** 2022-03-14

**Authors:** Qiutong Yu, Genyong Zuo

**Affiliations:** 1 Centre for Health Management and Policy Research, School of Public Health, Cheeloo College of Medicine Shandong University, 44 Wen-hua-xi Road, Jinan, 250012 Shandong China; 2 NHC Key Laboratory of Health Economics and Policy Research, Shandong University, 44 Wen-hua-xi Road, Jinan, 250012 Shandong China

**Keywords:** Indoor solid fuel, Blood pressure, Epidemiology, Hypertension, Indoor air pollution, Global health

## Abstract

Indoor air pollution caused by solid fuel use for cooking affects 2.5 billion people worldwide and may elevate blood pressure (BP) and increase the burden of hypertension. Although the elderly are the most at risk of an elevated BP and hypertension, few studies have evaluated the effect of indoor solid fuel use for cooking on BP in persons over the age of 65. Therefore, in this study, we randomly selected 8067 elderly people over 65 years of age from the 2018 Chinese Longitudinal Healthy Longevity Survey to determine the impact of indoor solid fuel use on BP/hypertension. The results showed that, compared with those who cooked with clean fuel, those who cooked with solid fuel had a 1.87 mmHg higher systolic blood pressure, a 0.09 mmHg higher diastolic blood pressure, a 0.97 mmHg higher pulse pressure, and a 1.22 mmHg higher mean arterial pressure. However, we did not find any association between indoor solid fuel use and hypertension. We further observed that northern China residents, women, people aged over 90 years, hypertensive and heart patients, and those with natural ventilation in the kitchen that used indoor solid fuel experienced a greater BP impact. Replacing solid fuel with clean fuel may be an important way to lower BP. Regarding this, priority access to clean fuel should be given to the susceptible population, including the elderly aged ≥ 75 years, northern China residents, women, and hypertensive and heart patients.

**Supplementary Information:**

The online version contains supplementary material available at 10.1007/s11356-022-19612-1.

## Introduction

Air quality is an important determinant of human health (Brauer et al. 2021). According to the World Health Organization, approximately 91% of the world’s population breathes polluted air. Air pollution is a man-made disaster that seriously affects the quality of human life and disability adjusted life years (Cohen et al. 2017). It is estimated that environmental air pollution causes 4.2 million deaths each year as a result of strokes, heart disease, lung cancer, and chronic respiratory diseases (World Health Organization 2021). Among these mortalities, nearly 2.8 million people died as a result of indoor air pollution caused by the use of solid fuel (Cohen et al. 2017).

In 2010, indoor air pollution from solid fuel use negatively impacted the global disease burden (Lim et al. 2012). Globally, approximately 2.5 billion people have been exposed to indoor air pollution from cooking with solid fuel (Bonjour et al. 2013). Burning solid fuel such as coal, wood, or crop residues can produce a large amount of particulate matter (PM_2.5_ and PM_10_), carbon monoxide, nitrogen dioxide, sulfur dioxide, and other volatile organic compounds, which are associated with blood pressure (BP) and hypertension (Everson et al. 2019; Liu et al. 2017; Wang et al. 2017; Yan et al. 2020; Yang et al. 2019). Hypertension, which is a main risk factor for stroke, heart disease, and renal failure (Stanaway et al. 2018), has caused 10.8 million deaths, accounting for 9.3% of disability-adjusted life years lost globally (IHME 2019a, b). Meanwhile, a systolic blood pressure (SBP) of at least 110 mmHg is associated with a variety of cardiovascular and renal diseases, including ischemic heart disease, cerebrovascular disease, and chronic kidney disease (Wright Jr et al. 2002; Collins et al. 1990). Further, a positive association between diastolic blood pressure (DBP), pulse pressure (PP), and mean arterial pressure (MAP) and the risk of cardiovascular disease is common in older individuals (Chobanian et al. 2003; Sesso et al. 2000). Therefore, it is necessary to explore the impact of indoor air pollution caused by solid fuel use on BP and hypertension.

In recent years, several studies have investigated the relationship between solid fuel use and BP and hypertension (Arku et al. 2018, 2020; Fatmi et al. 2019; Khan et al. 2021). However, the entire population was considered in these studies, including women, chefs, and patients with chronic diseases. One study examined 137,809 individuals aged 30–70 years from 640 urban and 21 rural areas and found that exposure to solid fuel pollution was negatively associated with BP and hypertension (Arku et al. 2020), but the average age of the study population was only 56 years. Three cross-section studies have evaluated the relationship between women using solid fuels and BP and hypertension, but they neglected the impact on men and had small sample sizes. A study of 77,605 premenopausal women aged 15–49 years from ten countries (Arku et al. 2018) and another study of 6543 women aged > 18 years conducted in Bangladesh demonstrated that women using solid fuels were more likely to suffer from hypertension (Khan et al. 2021). Conversely, a study of 850 women aged > 40 years conducted in Pakistan found no association between hypertension and indoor solid fuel use (Fatmi et al. 2019). Overall, there is limited research on whether the use of solid fuel by the elderly (aged greater than 65 years), which is the population most at risk to a raised BP, had an effect on BP and hypertension (Deng et al. 2020; Lin et al. 2021).

Currently, China’s average age is on an upward trend, wherein the elderly population has reached 190.64 million, accounting for 13.50% of the total population (National Bureau of Statistics of China 2021). To date, approximately 450 million Chinese still use solid fuel for cooking (Yu et al. 2018). Thus, it is necessary to explore the relationship between indoor solid fuel use and BP in the elderly population (Gall et al. 2013; Ji 2018). In this study, we explored the effect of using solid fuel for cooking on BP and hypertension using data from the Chinese Longitudinal Healthy Longevity Survey (CLHLS) by conducting a large population-based study including approximately 16,000 individuals aged ≥ 65 years in 500 urban and rural communities in 23 provinces in China.

## Methods

### Study population

In this study, we used secondary data from the 2018 CLHLS, which was a longitudinal population-based study of people older than 65 years in China. We used these data because they are the most recent available, and therefore, best fit China’s current population situation. Using a multi-stage stratified proportional probability sampling design, approximately 16,000 elderly people in urban and rural communities were randomly selected from 500 sample areas in 23 provinces. The biomedical ethics committee of Peking University approved the study, and all study participants signed an informed consent form. In total, 8067 individuals were found eligible for this study, excluding 95 participants younger than 65 years, 117 participants who answered “never cook,” 456 participants who refused to answer, 1095 participants who answered “not applicable,” 5990 participants with missing data (Fig. S1).

### BP measurement

After a study participant rested for at least 5 min, a research assistant measured the BP of the right arm twice using a mercury sphygmomanometer. For bedridden participants, BP was measured in the lying position. The interval between the two measurements was at least 1 min. For the SBP and DBP, we took the mean of the two measurements.

We defined hypertension patients as those with a self-reported history of hypertension, an average SBP ≥ 140 mmHg or an average DBP ≥ 90 mm Hg (World Health Organization 2013). Meanwhile, we defined PP as SBP-DBP and mean arterial pressure (MAP) as PP/3 + DBP to explore the possible mechanism affecting BP (Sesso et al. 2000). SBP, DBP, PP, and MAP were all defined as continuous variables, whereas the prevalence of hypertension was a binary variable.

### Indoor solid fuel use assessment

We determined the solid fuel used for cooking by asking the question, “What is the main source of cooking fuel in your family?” Participants who answered “other” were excluded from this research. Overall, we divided participants into two groups: (1) indoor solid fuel use, including coal, charcoal, and wood; and (2) clean fuel use, which includes solar energy, natural gas, induction cooker, and other electrical appliances.

### Covariates

We adjusted for numerous covariates according to previous studies (Arku et al. 2020; Hystad et al. 2019; Yan et al. 2016; Yao et al. 2021). Specifically, we adjusted for demographic characteristics, including age (years), gender (female/male), marital status (Married: married and living together/Not married: widowed, divorced, separated, or never married), residence (urban/rural), socioeconomic status, including education and family income, and cardiovascular disease (CVD) risk factors, including smoking status (no/yes, the answers “smoking now” or “in the past” were recognized as yes), alcohol use (no/yes), regular exercise (no/yes), body mass index (BMI; < 24 kg/m^2^/ ≥ 24 kg/m^2^) (Chittaranjan and Yajnik 2004), dietary diversity index (DDI; high: 8–9/median: 6–7/low: < 5) (Gina et al. 2010), and use of BP lowering medication (no/yes). All self-reported information was collected by well-trained researchers via in-person family interviews.

### Statistical analyses

Descriptive statistical data of fuel types among adults from urban and rural areas were reported proportionally, and the corresponding p-values were used to check whether there were statistically significant differences among these adults. Continuous demographic and socioeconomic variables were estimated by means of averages, and categorical variables by proportions. The chi-square test was used for dichotomous variables, and the t-test was used for continuous variables.

The relationship between solid fuel for cooking and BP/hypertension was assessed using a multivariable regression model. The first crude model was unadjusted. Then, we specified a base model (Model 1) that included age, gender, urban/rural status, and marital status, after which groups of CVD risk factors were progressively added. Model 2 adjusted for additional risk factors, including smoking status, alcohol use, physical activity, BMI, dietary patterns, and use of BP lowering medication. The fully adjusted model (Model 3) included Model 2 covariates as well as socioeconomic status, including years of education and family income.

Subgroup analyses of the association of household solid cooking fuel with hypertension prevalence or BP were performed according to demographic characteristics and CVD risk factors using a multivariable regression model and multiple interactions. Demographic characteristics included region (south/north, in which the provinces of southern China included Shanghai, Jiangsu, Zhejiang, Anhui, Fujian, Jiangxi, Hubei, Hunan, Guangdong, Guangxi, Hainan, Chongqing, and Sichuan; and those of northern China included Beijing, Tianjin, Hebei, Shanxi, Liaoning, Jilin, Heilongjiang, Shandong, Henan, and Shaanxi), residence (urban/rural), gender, age (elderly age/senile age/long-livers; for which the age of participants was categorized into three groups according to the age standards revised by the World Health Organization in 2015: elderly age 60–75 years, senile age 75–90 years, and long-livers older than 90 years (Prusinowska et al. 2016)), indoor ventilation (none/natural/mechanical, for which participants answering “no ventilation measures” were considered as none, “naturally open windows for ventilation” were considered natural, and “range hood” and “exhaust fan” were classified as mechanical). CVD risk factors included the BMI (< 24 kg/m^2^/ ≥ 24 kg/m^2^), hypertension (no/yes), anti-hypertension medication use (no/yes), diabetes mellitus (no/yes), and heart diseases (no/yes).

## Results

### Basic participant characteristics

The characteristics of the study participants according to their indoor solid fuel cooking use are summarized in Table 1. Participants from south and north China made up 68.32% and 31.68% of the total study population, respectively. The mean age of all participants was 84.37 years. In urban areas, 21.47% of participants used indoor solid fuel for cooking, whereas in rural areas, this proportion reached 41.53%. Urban residents that used solid fuel (138.62 mmHg/80.19 mmHg) had both a higher SBP and DBP (*P* < 0.001) as compared to those who used clean fuel (136.58 mmHg/78.27 mmHg), as were rural residents, showing that those who use solid fuel for cooking are at a higher risk of an elevated BP. Moreover, participants who use clean fuel had higher socioeconomic indicators, both in terms of years of education and family income, than those using indoor solid fuel.

**Table 1:** Characteristics of 8067 individuals with solid fuel use for cooking, stratified by urban/rural status and solid fuel use for cooking versus clean fuel

Characteristic	All participants	Urban (*n* = 4523)			Rural (*n* = 3544)		
		Solid fuel	Clean fuel	*P*	Solid fuel	Clean fuel	*P*
Individuals (n, %)	8067	971 (21.47)	3,552 (78.53)		1,472 (41.53)	2,072 (58.47)	
SBP (mmHg) (mean, SD)	138.62 (20.79)	138.62 (21.46)	136.58 (19.61)	< 0.001	141.64 (22.08)	139.98 (21.14)	0.024
DBP (mmHg) (mean, SD)	79.32 (11.74)	80.19 (11.53)	78.27 (11.64)	< 0.001	80.62 (12.36)	79.77 (11.38)	0.035
PP (mmHg) (mean, SD)	59.30 (18.08)	58.43 (18.26)	58.30 (17.91)	0.841	61.02 (18.21)	60.21 (18.10)	0.190
MAP (mmHg) (mean, SD)	99.09 (12.78)	99.66 (12.97)	97.71 (12.14)	< 0.001	100.96 (13.81)	99.84 (12.75)	0.013
Hypertension (n, %)				0.001			0.138
No	2,998 (37.16)	410 (42.22)	1,283 (36.12)		563 (38.25)	742 (35.81)	
Yes	5,069 (62.84)	561 (57.78)	2,269 (63.88)		909 (61.75)	1,330 (64.1)	
Region (n, %)				0.163			< 0.001
South	5,511 (68.32)	665 (68.49)	2,348 (66.10)		932 (63.32)	1,566 (75.58)	
North	2,556 (31.68)	306 (31.51)	1,204 (33.90)		540 (36.68)	506 (24.42)	
Age (years, mean, SD)	84.37 (11.82)	84.28 (11.83)	84.55 (11.78)	0.528	84.27 (11.76)	84.18 (11.95)	0.746
Sex (n, %)				0.173			0.263
Female	4,457 (55.25)	540 (55.61)	1,888 (53.15)		859 (58.36)	1,170 (56.47)	
Male	3,610 (44.75)	431 (44.39)	1,664 (46.85)		613 (41.64)	902 (43.53)	
Marital status (n, %)				0.302			0.056
Not Married	4,446 (55.11)	515 (53.04)	1,950 (54.90)		795 (54.01)	1,186 (57.24)	
Married	3,621 (44.89)	456 (46.96)	1,602 (45.10)		677 (45.99)	886 (42.76)	
Education (years, mean, SD)	3.33 (4.13)	2.00 (2.95)	4.59 (4.79)	< 0.001	2.09 (2.94)	2.68 (3.46)	< 0.001
Income (yuan, mean, SD)	41,687.28 (36,244.09)	24,439.89 (28,362.57)	56,120.33 (35,899.06)	< 0.001	22,010.39 (26,988.49)	39,006.51 (35,118.00)	< 0.001
Smoking status (n, %)				0.925			0.030
No	5,537 (68.64)	665 (68.49)	2,427 (68.33)		1,045 (70.99)	1,400 (67.57)	
Yes	2,530 (31.36)	306 (31.51)	1,125 (31.67)		427 (29.01)	672 (32.43)	
Alcohol use (n, %)				0.046			0.054
No	5,879 (72.88)	682 (70.24)	2,609 (73.45)		1,100 (74.73)	1,488 (71.81)	
Yes	2,188 (27.12)	289 (29.76)	943 (26.55)		372 (25.27)	584 (28.19)	
Exercise regularly (n, %)				< 0.001			< 0.001
No	4,750 (58.88)	722 (74.36)	1,563 (44.00)		1,091 (74.12)	1,374 (66.31)	
Yes	3,317 (41.12)	249 (25.64)	1,989 (56.00)		381 (25.88)	698 (33.69)	
BMI (n, %)				< 0.001			0.003
< 24 (kg/m^2^)	5,480 (67.93)	724 (74.56)	2,267 (63.82)		1,074 (72.96)	1,415 (68.29)	
≥ 24 (kg/m^2^)	2,587 (32.07)	247 (25.44)	1,285 (36.18)		398 (27.04)	657 (31.71)	
Dietary diversity^a^ (n, %)				< 0.001			< 0.001
Low	1,425 (17.66)	264 (27.19)	416 (11.71)		354 (24.05)	391 (18.87)	
Median	3,185 (39.48)	444 (45.73)	1,190 (33.50)		645 (43.82)	906 (43.73)	
High	3,457 (42.85)	263 (27.09)	1,946 (54.79)		473 (32.13)	775 (37.40)	
Anti-hypertension medication (n, %)				< 0.001			< 0.001
No	4,848 (60.10)	638 (65.71)	1,980 (55.74)		978 (66.44)	1,252 (60.42)	
Yes	3,219 (39.90)	333 (34.29)	1,572 (44.26)		494 (33.56)	820 (39.58)	
Indoor ventilation (n, %)				< 0.001			< 0.001
None	696 (8.66)	151 (15.66)	177 (4.99)		200 (13.73)	168 (8.14)	
Natural	3,784 (47.11)	715 (74.17)	828 (23.34)		1,147 (78.72)	1,094 (52.98)	
Mechanical	3,553 (44.23)	98 (10.17)	2,542 (71.67)		110 (7.55)	803 (38.89)	
Diabetes (n, %)				< 0.001			< 0.001
No	6,639 (85.38)	824 (88.70)	2,806 (82.05)		1,285 (90.43)	1,724 (85.94)	
Yes	1,137 (14.62)	105 (11.30)	614 (17.95)		136 (9.57)	282 (14.06)	
Heart disease (n, %)				< 0.001			< 0.001
No	6,194 (79.69)	782 (84.09)	2,601 (76.10)		1,193 (83.95)	1,618 (80.74)	
Yes	1,579 (20.31)	148 (15.91)	817 (23.90)		228 (16.05)	386 (19.26)	

*SBP*, systolic blood pressure; *DBP*, diastolic blood pressure; *BMI*, body mass index; *SD*, standard deviation

^a^Dietary diversity: Low (Dietary Diversity Index [DDI] ≤ 4); median (DDI: 5–6); high (DDI: ≥ 7)

### Distribution of indoor solid fuel use across provinces

**Fig. 1:**
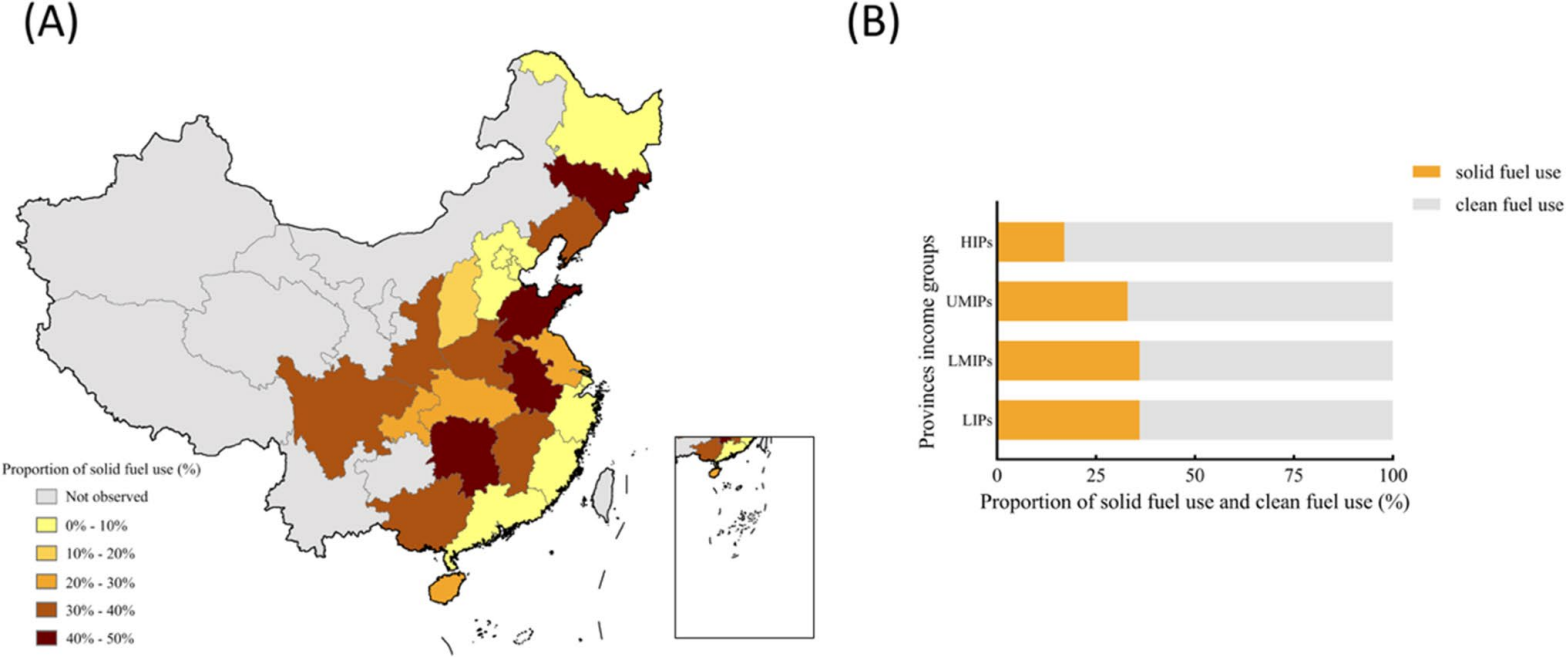
Proportion of solid fuel used by province of China based on 2018 CLHLS (**A**); percentage chart shows the proportion of solid fuel use classified by per capita disposable income of provinces (**B**). High-income provinces (HIPs): Beijing, Jiangsu, Shanghai, Tianjin, Zhejiang; Upper Middle-income provinces (UMIPs): Fujian, Guangdong, Liaoning, Shandong, Chongqing; Lower Middle-income provinces (LMIPs): Anhui, Hainan, Hebei, Heilongjiang, Hubei, Hunan, Jilin, Jiangxi, Shaanxi; Low-income provinces (LIPs): Guangxi, Henan, Shanxi, Sichuan. The economic level classification of a province is based on the interquartile range of the per capita disposable income of each province released by the National Bureau of Statistics in 2018

Figure 1 shows the location of the study communities, the proportion of cooking with indoor solid fuel in each province (Fig. 1A), and the per capita distribution of resident disposable income in an interquartile range (Fig. 1B). As shown in Fig. 1A, less than 10% of the population residing in the provinces of Beijing, Fujian, Guangdong, Hebei, Heilongjiang, Shanghai, Tianjin, and Zhejiang used solid fuel, whereas in Anhui, Hunan, Jilin, Jiangxi, and Shandong, nearly 50% of the population used indoor solid fuel. As shown in Fig. 1B, low-income and lower middle-income provinces had the highest solid fuel exposure, wherein the proportion of people using solid fuel reached 36%. Conversely, high-income provinces had the lowest proportion of cooking with indoor solid fuel, at only 17%.

### Association of indoor solid fuel use with BP and hypertension

Table 2 summarizes the association between indoor solid fuel use and BP. The unadjusted Model and Model 1 revealed that solid fuel use for cooking is strongly associated with an increased BP. Model 1 also showed that people who cooked primarily with indoor solid fuel had a lower likelihood of hypertension (OR = 0.83; 95% CI: 0.75, 0.92). Model 2, which included CVD risk factors, produced the same results as Model 1, but showed an increased effect of solid fuel on BP. Moreover, Model 3, which included socioeconomic status measures, revealed a weaker association between solid fuel use and BP/hypertension. Overall, compared with those who cooked with clean fuel, those who cooked with solid fuel had a 1.87 mmHg higher SBP (95% CI: 0.85, 2.89), a 0.09 mmHg higher DBP (95% CI: 0.31, 1.49), a 0.97 mmHg higher PP (95% CI: 0.06, 1.88), and a 1.22 mmHg higher MAP (95% CI: 0.59, 1.85). However, there was no significant association with hypertension (OR = 0.99, 95% CI: 0.87, 1.14).

**Table 2:** Associations between use of solid fuels for cooking, versus clean fuels, and BP parameters and the odds of having hypertension

	SBP β (95% CI)	DBP β (95% CI)	PP β (95% CI)	MAP β (95% CI)	Hypertension OR (95% CI)
Crude model	2.60 (1.62, 3.59) ***	1.62 (1.06, 2.18) ***	0.99 (0.13, 1.85) *	1.95 (1.34, 2.55) ***	0.85 (0.77, 0.94) **
Model 1	1.82 (0.81, 2.82) ***	1.35 (0.79, 1.92) ***	0.46 (-0.41, 1.34)	1.51 (0.89, 2.13) ***	0.83 (0.75, 0.92) ***
Model 2	2.72 (1.73, 3.70) ***	1.63 (1.06, 2.20) ***	1.09 (0.21, 1.96) *	1.99 (1.38, 2.60) ***	1.05 (0.92, 1.19)
Model 3	1.87 (0.85, 2.89) ***	0.90 (0.31, 1.49) **	0.97 (0.06, 1.88) *	1.22 (0.59, 1.85) ***	0.99 (0.87, 1.14)

Model 1: age, gender, urban/rural status, marital status; Model 2: Model 1 + smoking status, alcohol use, physical activity, BMI, dietary diversity, use of BP lowering medication; Model 3: Model 2 + education, income

*OR*, odds ratio; *SBP*, systolic blood pressure; *DBP*, diastolic blood pressure, PP, MAP*** *p* < 0.001, ** *p* < 0.01, * *p* < 0.05

### Subgroup analyses of indoor solid fuel use with BP and hypertension

Table 3 lists the results of the stratified analysis and interaction effect. Regarding geographic location, we found that the SBP of residents using solid fuel in north China (95% CI: 0.13, 1.64) was 0.25 mmHg higher than that of residents in south China (95% CI: 0.00, 2.28), for which the interaction term was significant (*p* < 0.001). Meanwhile, in terms of age, the results showed that long-livers who cooked with solid fuel had a SBP 3.37 mmHg (95% CI: 1.57, 5.17) higher than those who cooked with clean fuel, and that there was a significant interaction term (*p* < 0.001). It was found that for women older than 65 years, using solid fuel greatly impacted BP (*β* = 2.76 mmHg, 95% CI: 1.32, 4.19 for SBP; *β* = 1.08 mmHg, 95% CI: 0.27, 1.89). In addition, heart disease patients using indoor solid fuel had a 1.57 mmHg higher DBP than those who cooked with clean fuel (95% CI: 0.10, 3.04). Regarding indoor ventilation, we observed a strong association between indoor solid fuel use for cooking and an increased SBP/DBP. Specifically, if a kitchen was naturally ventilated when cooking, people who cooked with solid fuel had an SBP 1.81 mmHg (95% CI: 0.47, 3.15) and a DBP 1.18 mmHg (CI: 0.43, 1.93) higher than those who cooked with clean fuel. Nevertheless, we did not find that using indoor solid fuel was related to BP or hypertension in the case of those using mechanical ventilation and those with no ventilation.

**Table 3:** Model results for multivariable analysis of the association of BP parameters and odds of hypertension with solid fuel use, stratified by selected variables

Entire sample	N	Systolic BP β (95% CI)	Diastolic BP β (95% CI)	PP β (95% CI)	MAP β (95% CI)	Hypertension OR (95% CI)
	8067	1.87 (0.85, 2.89)	0.90 (0.31, 1.49)	0.97 (0.06, 1.88)	1.22 (0.59, 1.85)	0.99 (0.87, 1.14)
Region						
South	5511	0.67 (-0.56, 1.89)	1.00 (0.31, 1.70)	-0.33 (-1.39, 0.73)	0.89 (0.13,1.64) *	0.91 (0.77, 1.07)
North	2556	3.50 (1.65, 5.35) ***	-0.03 (-1.16,1.08)	3.54 (1.79, 5.29)	1.14 (0.00, 2.28) *	1.12 (0.87, 1.44)
Interaction effect		3.40 (1.88, 4.92) ***	1.67 (0.82, 2.53) ***	1.72 (0.39, 3.06) *	2.25 (1.32, 3.18) ***	1.06 (0.91, 1.25)
Community Location						
Urban	4523	1.49 (-0.00, 2.99)	0.78 (-0.11, 1.66)	0.72 (-0.65, 2.09) *	1.01 (0.09, 1.94) *	0.89 (0.72, 1.10)
Rural	3544	2.07 (0.65, 3.49) **	0.94 (0.15, 1.73) *	1.13 (-0.10, 2.35)	1.32 (0.45, 2.19) **	1.06 (0.89, 1.27)
Interaction effect		1.53 (-0.02, 3.09)	1.14 (0.26, 2.01) *	0.40 (-0.97, 1.76)	1.27 (0.32, 2.22) **	0.91 (0.78, 1.06)
Gender						
Female	4457	2.76 (1.32, 4.19) ***	1.08 (0.27, 1.89) **	1.68 (0.37, 2.99) *	1.64 (0.77, 2.50) ***	1.00 (0.84, 1.20)
Male	3610	0.71 (-0.72, 2.13)	0.68 (-0.17, 1.54)	0.02 (-1.20, 1.25)	0.69 (-0.22, 1.60)	0.99 (0.80, 1.21)
Interaction effect		0.16 (-1.38, 1.71)	0.27 (-0.60, 1.13)	-0.11 (-1.46, 1.25)	0.23 (-0.71, 1.18)	0.92 (0.79, 1.08)
Age						
Elderly age	2056	-0.40 (-2.26, 1.46)	0.22 (-0.85, 1.30)	-0.62 (-2.20, 0.96)	0.02 (-1.16, 1.19)	0.88 (0.66, 1.16)
Senile age	2992	1.94 (0.32, 3.56) *	0.80 (-0.20, 1.79)	1.14 (-0.36, 2.64)	1.18 (0.16, 2.20) *	0.86 (0.68, 1.10)
Long-livers	3019	3.37 (1.57, 5.17) ***	1.53 (0.54, 2.52) **	1.84 (0.25, 3.44) *	2.14 (1.06, 3.22) ***	1.15 (0.94, 1.41)
Interaction effect		0.86 (0.39, 1.32) ***	0.43 (0.17, 0.69) **	0.43 (0.03, 0.84) *	0.57 (0.29, 0.85) ***	0.99 (0.95, 1.04)
BMI						
< 24 (kg/m^2^)	5480	1.67 (0.45, 2.90) **	0.76 (0.06, 1.46) *	0.92 (-0.19, 2.02)	1.06 (0.32, 1.81) **	0.88 (0.76, 1.04)
≥ 24 (kg/m^2^)	2587	2.53 (0.68, 4.38) **	1.32 (0.22, 2.42) *	1.21 (-0.41, 2.83)	1.72 (0.56, 2.89) **	1.48 (1.11, 1.96) **
Interaction effect		2.95 (1.17, 4.72) **	1.98 (0.98, 2.97) ***	0.97 (-0.58, 2.52)	2.30 (1.22, 3.38) ***	1.28 (1.05, 1.57) *
Hypertension						
No	2998	0.45 (-0.49, 1.40)	0.20 (-0.49, 0.90)	0.25 (-0.61, 1.11)	0.29 (-0.38, 0.96)	NA
Yes	5069	2.80 (1.50, 4.10) ***	1.40 (0.60, 2.19) **	1.41 (0.15, 2.67) *	1.86 (1.07, 2.66) ***	NA
Interaction effect		15.08 (13.92, 16.25) ***	5.73 (5.06, 6.40) ***	9.36 (8.32, 10.40) ***	8.85 (8.14, 9.56) ***	NA
Anti-hypertension medication use						
No	4848	0.64 (-0.59, 1.87)	0.45 (-0.22, 1.13)	0.19 (-0.88, 1.26)	0.51 (-0.23, 1.26)	1.00 (0.87, 1.14)
Yes	3219	4.28 (2.51, 6.06) ***	1.91 (0.82, 3.00) **	2.38 (0.73, 4.02) **	2.70 (1.59, 3.82) ***	NA
Interaction effect		5.09 (3.48, 6.70) ***	2.73 (1.80, 3.66) ***	2.36 (0.93, 3.80) **	3.52 (2.53, 4.50) ***	NA
Indoor ventilation						
None	696	0.01 (-3.01, 3.03)	-0.17 (-1.87, 1.53)	0.18 (-2.44, 2.80)	-0.11 (-1.97, 1.75)	1.26 (0.83, 1.90)
Natural	3784	1.81 (0.47, 3.15) **	1.18 (0.43, 1.93) **	0.63 (-0.54, 1.79)	1.39 (0.57, 2.21) **	0.95 (0.80, 1.13)
Mechanical	3553	0.04 (-2.65, 2.74)	-0.67 (-2.30, 0.95)	0.72 (-1.78, 3.22)	-0.43 (-2.11, 1.24)	0.72 (0.49, 1.07)
Interaction effect		0.61 (0.08, 1.13) *	0.28 (-0.01, 0.59)	0.32 (-0.14, 0.78)	0.39 (0.07, 0.71) *	0.95 (0.90, 1.01)
Diabetes mellitus						
No	6639	1.96 (0.84, 3.07) **	0.87 (0.23, 1.50) **	1.09 (0.10, 2.08) *	1.23 (0.55, 1.91) ***	0.99 (0.86, 1.14)
Yes	1137	0.25 (-2.68, 3.25)	1.17 (-0.61, 2.95)	-0.88 (-3.63, 1.87)	0.87 (-0.96, 2.70)	1.70 (0.93, 3.08)
Interaction effect		0.37 (-2.58, 3.32)	2.01 (0.36, 3.66) *	-1.64 (-4.23, 0.95)	1.46 (-0.33, 3.26)	1.76 (1.08, 2.88) *
Heart disease						
No	6194	1.57 (0.42, 2.72) **	0.71 (0.06, 1.36) *	0.86 (-0.16, 1.89)	1.00 (0.30, 1.70) **	0.97 (0.84, 1.13)
Yes	1579	2.29 (-0.15, 4.74)	1.57 (0.10, 3.04) *	0.72 (-1.52, 2.97)	1.81 (0.29, 3.34) *	1.12 (0.78, 1.61)
Interaction effect		1.70 (-0.71, 4.12)	1.73 (0.38, 3.08) *	-0.03 (-2.15, 2.10)	1.72 (0.25, 3.19) *	0.94 (0.71, 1.25)

*BP*, blood pressure; *BMI*, body mass index. ^***^p < 0.001, ^**^p < 0.01, ^*^p < 0.05

### Meta-analysis of regional impact on indoor air pollution

**Fig. 2:**
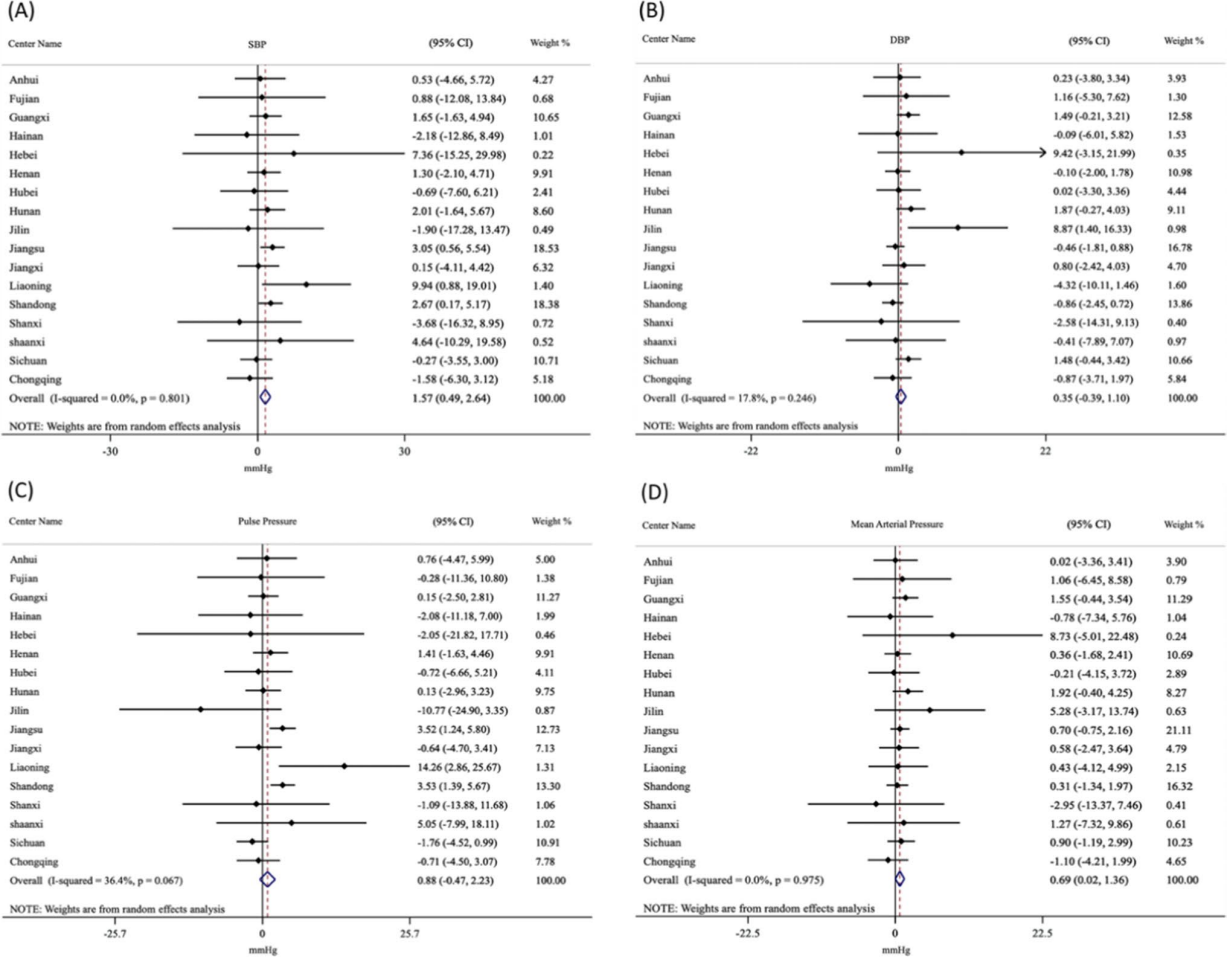
Model estimates of multivariable, province-specific, and meta-analysis of the association of solid fuel use with (**A**) SBP; (**B**) DBP; (**C**) PP; and (**D**) MAP based on 2018 CLHLS

Finally, the association between solid fuel use for cooking and BP was explored using a meta-analysis according to geographic location. As shown in Fig. 2, the overall pooled β for SBP was 1.57 (95 CI%: 0.49, 2.64) with *I*^2^ = 0 and *P* = 0.80, that for DBP was 0.35 (95 CI%: -0.39, 1.10) with *I*^2^ = 17.8% and *P* = 0.25, that for PP was 0.88 (95 CI%: -0.47, 2.23) with *I*^2^ = 36.4% and *P* = 0.07, and that for MAP was 0.69 (95 CI%: 0.02, 1.36) with *I*^2^ = 0 and *P* = 0.98. Because of sample size limitations, we did not run province-specific models for Beijing, Guangdong, Heilongjiang, Shanghai, or Zhejiang.

## Discussion

### Summary of findings

The results of our cross-sectional study of 8067 elderly people over 65 years of age in 500 urban and rural communities in 23 provinces in China showed that people who used solid fuel for cooking may be at a greater risk of an elevated BP than those who used clean fuel. However, we did not find any significant association between solid fuel use for cooking and hypertension.

### Robustness of findings

Our findings verified existing evidence that the use of solid fuel for cooking by the elderly may be positively correlated with BP. Although a large number of older people are exposed to solid fuel pollution and may experience health effects, few studies have investigated the impact of indoor air pollution caused by solid fuel use for cooking for the elderly over the age of 65 years. Furthermore, only two studies have examined indoor solid fuel use for the elderly as related to BP, and these studies reached contradictory conclusions. A study of 3754 participants (≥ 45 years old) in China showed that people cooking with solid fuel were 1.15 times more likely to suffer from hypertension (OR = 1.15, 95% CI: 1.01, 1.31), and had an SBP 1.10 mmHg (95% CI: 0.48, 1.72), a DBP 1.02 mmHg (95% CI: 0.61, 1.43), and an MAP 1.03 mmHg (95% CI: 0.63, 1.43) higher than those who cooked with clean fuel (Deng et al. 2020). Another study of 10,450 participants (≥ 65 years old) in China did not reveal any significant association between indoor solid fuel use for cooking and BP or hypertension (Lin et al. 2021). In addition, two Urban and Rural Epidemiology (PURE) studies considered participants aged 30–70 years. One of these studies did not find that elderly people (≥ 50 years old) who use indoor solid fuel were more likely to suffer from hypertension or an increased BP (Arku et al. 2020), whereas a subgroup analysis in the other study showed that there was no association between solid fuel use and hypertension/BP in the elderly over 60 years (Liu et al. 2021). These studies demonstrate the variability of results related to indoor solid fuel use for cooking and BP/hypertension.

These varying results might be explained by variations in the sample size and insufficient covariates, especially CVD risk factors. In this study, we found that, compared with those who cooked with clean fuel, those who cooked with solid fuel had a 1.87 mmHg higher SBP (95% CI: 0.85, 2.89), a 0.09 mmHg higher DBP (95% CI: 0.31, 1.49), a 0.97 mmHg higher PP (95% CI: 0.06, 1.88), and a 1.22 mmHg higher MAP (95% CI: 0.59, 1.85). Our findings were robust across study regions, urban/rural status, CVD risk factors, and sociodemographic characteristics. Furthermore, our study had a large sample of older people over 65 years of age, in which the average age was 84.37 years.

### Reducing rise in BP caused by indoor solid fuel use

This is the first elderly-based multicenter study to investigate the association between indoor solid fuel use for cooking and BP/hypertension while controlling for comprehensive factors. The large proportion of elderly people in our study that had an elevated BP or suffered from hypertension may be the result of a lack of medical care. Model 3 considered education and family income, which may be a component of health care, and revealed the weakest impact of solid fuel on BP of all the models used. Specifically, the SBP, DBP, PP, and MAP values decreased from 2.72 mmHg (95% CI: 1.73, 3.70), 1.63 mmHg (95% CI: 1.06, 2.20), 1.09 mmHg (95% CI: 0.21, 1.96), and 1.99 mmHg (95% CI: 1.38, 2.60) in Model 2 to 1.87 mmHg (95% CI: 0.85, 2.89), 0.90 mmHg (95% CI: 0.31, 1.49), 0.97 mmHg (95% CI: 0.06, 1.88), and 1.22 mmHg (95% CI: 0.59, 1.85) in Model 3, respectively, the results of which are consistent with the large PURE study (Hystad et al. 2019). Many studies have demonstrated a positive association between SBP or DBP and the risk of cardiovascular CVD (Joint National Committee on the Detection 1997). PP is affected by the left ventricular ejection fraction and large arterial stiffness, representing changes in blood pressure. A high PP indicates a poor blood vessel elasticity (Franklin et al. 1997). Additionally, MAP has been extensively researched and shown to be positively associated with CVD in some studies (Benetos et al. 1997). Previous literature suggests that the underlying mechanisms of a BP increase with solid fuel use includes an increased inflammation and oxidative stress (Dutta et al. 2012), which subsequently leads to an increased BP (Dinh et al. 2014). Additionally, solid fuel in the combustion chamber produces toxic volatile organic compounds (VOCs), which can easily turn into vapors and are involved in metabolic processes that lead to an increased BP (Sun et al. 2019). Therefore, it may be biologically plausible that we observed a significant association between indoor air pollution and BP. Although we did not observe a significant positive association between indoor solid fuel use and hypertension in Model 3, our study included approximately 500 sample areas in 23 provinces in China, which may not be representative of all of China.

### Elderly over the age of 75 should prioritize replacing indoor solid fuel

Our study provided the novel finding that long-livers (*β* = 1.94 mmHg, 95% CI: 0.32, 3.56 for SBP; *β* = 1.18 mmHg, 95% CI: 0.16, 2.20 for MAP) may be at a greater risk of an increased BP than those of senile age among those using solid fuel for cooking (*β* = 3.37 mmHg, 95% CI: 1.57, 5.17 for SBP; *β* = 2.14 mmHg, 95% CI: 1.06, 3.22 for MAP). Age is a determining risk associated with an increased BP and hypertension (Peter and Paul 2017), and the results of the 2012–2015 Chinese Hypertension Study showed that the prevalence of hypertension was 55.7% in those aged 65–74 years, and 60.2% in those aged ≥ 75 years (Wang et al. 2018). Therefore, with increasing population life expectancy, the number of elderly hypertensive patients is also expected to increase. These results provide useful insights for countries that are experiencing an aging society like China. In the case of limited clean energy facilities or other human and material resources, priority should be given to the susceptible population, which is those aged over 75 years.

### Different groups should be given different attention

Stratified analysis showed that a significant association of solid fuel and blood pressure was found only in people with hypertension, but not in non-hypertensive people. This suggests that the pathogenic effect of solid fuel that leads to an elevated BP mainly exists in hypertensive patients. Additionally, consist with previous study (Arku et al. 2020), no pathogenic effects of solid fuel were found in this research when BP was normal. The possible reason for this is that this study only included people over 65 years of age. According to the researching reported that the prevalence of hypertension decreased with age in people over 65 years old (Lu et al. 2017). The population over 65 years of age without hypertension in this study may be insensitive to the prevalence of hypertension. According to the stratified analyses, use of indoor solid fuel for cooking by northern residents might have a greater impact on BP than that by southern residents. One possible explanation for this is that the long-term exposure of northern residents to PM_2.5_, combined with the harmful gases released by using indoor solid fuel, increased the BP of northern residents (Dong et al. 2013; Li et al. 2018). Notably, we found a significant association for women over the age of 65 using solid fuel for cooking, but not for men. In most Chinese households, women are the primary cooks, meaning that elderly women are exposed to air pollution for a longer time than men, giving them higher exposure levels (Austin and Mejia 2017; Ofori et al. 2018). As the results showed, the elderly over the age of 75 were most affected by solid fuel use. In addition, the BP of the elderly suffering from hypertension and heart disease was significantly related to the use of indoor solid fuel, which is consistent with some previous studies (Dutta et al. 2011; Misun et al. 2012), but contrary to others (Arku et al. 2020). We also found that natural ventilation had the greatest impact on BP. However, we did not observe any association with mechanical ventilation and no ventilation. This is likely because there were only 696 no ventilation cases documented in the data, and mechanical ventilation had the best ventilation effect. The magnitude of these differences was consistent with previous findings concerning the impact of ventilation (Rehfuess et al. 2011).

### Strengths and limitations

Our study has several strengths, including the large sample size from 500 urban and rural communities across 23 provinces in China, making it a useful supplement to the elderly group data for future studies (Arku et al. 2020; Liu et al. 2021). Furthermore, the inclusion of multiple provinces and communities increased the generality of the results, the extensive individual information collected aided to control confusion, and standardizing and defining comprehensive and systematic information helped to evaluate the results. However, there were also some limitations. First, a limitation of our study is that we cannot make causal inferences since this study is cross-sectional in nature. Second, we had no information regarding whether the individuals were responsible for cooking, what types of cooking they experienced during their childhood, and how much time they spent cooking with indoor solid fuel. In addition, indoor air pollution exposure may vary as a result of familial and personal characteristics (Balakrishnan et al. 2011). Third, we did not have the capacity to evaluate specific fuel types (for example, manure, wood, and agricultural products), and additional analyses are required in future research. Fourth, we used indoor solid fuel for cooking as the only indicator of indoor air pollution exposure because we did not know the exact residential addresses of participants; therefore, we did not collect personal level exposure PM_2.5_ data (Adar et al. 2018; Zhang et al. 2019). Fifth, our study did not include temperature and humidity, both of which impact BP and hypertension (Lewington et al. 2012; Modesti 2013) because the long-term temperature and humidity of the respective households were not measured in the survey.

## Conclusions

We found that, compared with households that use clean fuel for cooking, elderly persons living in households that use indoor solid fuel for cooking might have an increased risk of and elevated BP. The results from this large, diverse population of 8067 elderly people aged over 65 years from 500 urban and rural communities in 23 provinces in China support the notion that solid fuel use for cooking may be positively related to BP. On this basis, replacing solid fuel for cooking with clean fuel may be an important way to lower BP, and the most susceptible population, those aged over 75 years, northern residents, women, and hypertensive and heart patients, should be given priority access to clean fuels.

## Supplementary Information

Below is the link to the electronic supplementary material.

Supplementary file1 (DOCX 247 kb)

## Data Availability

Data are available on the open research data service platform of Peking University. Data for this study were sourced from the Chinese Longitudinal Healthy Longevity Survey (CLHLS) and are available here: https://opendata.pku.edu.cn.
