# Supplementary material for: Relationship of indoor solid fuel use for cooking with blood pressure and hypertension among the elderly in China
Source: Environ Sci Pollut Res Int. 2022 Mar 14;29(35):53444–55. doi: 10.1007/s11356-022-19612-1 (PMC9343286; doi:10.1007/s11356-022-19612-1)
Supplement: Supplementary file 1 — Supplementary file1 (DOCX 247 kb) [file 11356_2022_19612_MOESM1_ESM.docx]

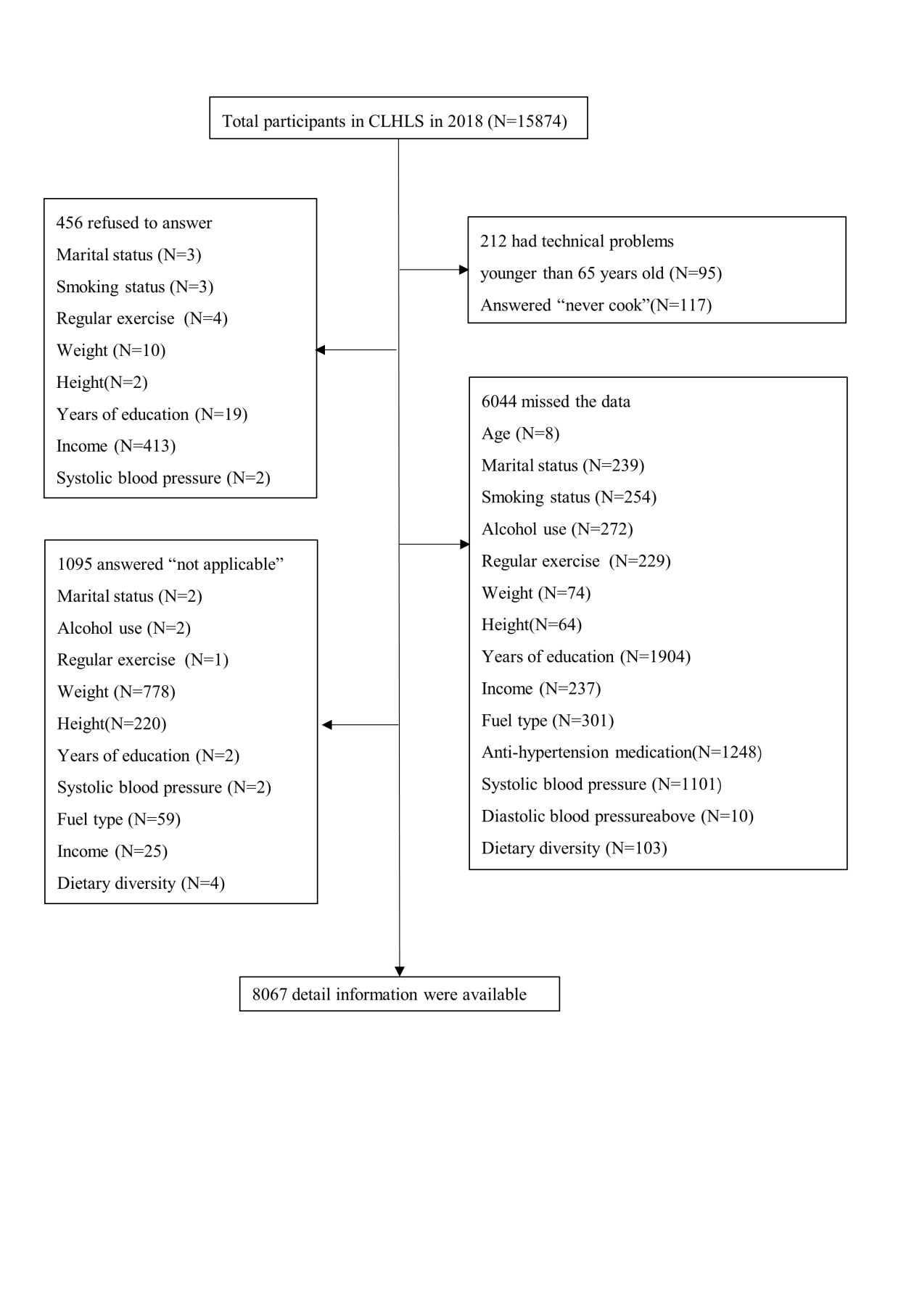


Figure S1 Study flowchart of participants selection (aged 65years or over) from Chinese Longitudinal Healthy Longevity Survey 2018 survey data.
